# Two new and rare mountain door-snails (Gastropoda, Pulmonata, Clausiliidae) from high mountain areas in Macedonia

**DOI:** 10.3897/zookeys.168.1919

**Published:** 2012-01-31

**Authors:** Ivaylo Kanev Dedov

**Affiliations:** 1 Institute of Biodiversity and Ecosystems Research, Gagarin Str., 1113 Sofia, Bulgaria

**Keywords:** new species, *Euxinella alpinella* sp. n., *Vestia lazarovii* sp. n., variation of clausilium, Republic of Macedonia

## Abstract

Two species of Clausiliidaeare described as new to science. *Euxinella alpinella* **sp. n.** is the fourth species within genus *Euxinella* Nordsieck, 1973, and *Vestia lazarovii* **sp. n.** is the second species of genus *Vestia* recorded from the Republic of Macedonia. In both species, the clausilium apparatus shows a high degree of variation.

## Introduction

Until recently, only one species of the genus *Euxinella* with its locus typicus in the Bistra Mountains in Republic of Macedoniawas known: *Euxinella radikae radikae* Nordsieck, 1973. Dedov and Neubert (2009) expanded the known distribution of the nominotypical subspecies to the Jablanitsa Mountains (Republic of Macedonia) and published a new subspecies, *Euxinella radikae hristovskii* Dedov & Neubert, 2009 from the Gyonovitsa Cave, Bukovich Hill (Republic of Macedonia). They also described a new species *Euxinella subaii* Dedov & Neubert, 2009, from the Mihailovo Mountain Resort in the Kozhuf Mountains (Republic of Macedonia). *Euxinella subaii* shows some shell morphological characters that are not conform to the generic definition of *Euxinella* (Nordsieck 1973) as for example a very long lamella subcolumellaris. It would be desirable to investigate the morphology of the genital system of all meanwhile known representatives of *Euxinella* in order to improve the generic diagnosis (cf. Dedov and Neubert 2009). *Euxinella alpinella* sp. n. is the second species within the genus with a long subcolumellaris, and thus is probably close to *Euxinella subaii*.

The genus *Vestia* was mentioned for the first time from the Republic of Macedonia by Urbanski (1960) from Osogovo Mountains, Kalin Kamen area, 1560 m a.s.l. with *Vestia* (*Brabenecia*) *ranojevici ranojevici* (Pavlovic 1912), which was later confirmed by H. Nordsieck (1974). Dedov (2010) added another record of this subspecies from a new site in the Osogovo Mountains (particularly from the Bulgarian part of the mountains).

## Material and methods

Most of the material was collected by the author in 2009 and 2010 from the Nidzhe and Baba Mountains, Republic of Macedonia. The first specimens of *Vestia lazarovii* sp. n. were collectedby Dr. Stoyan Lazarov in 2002, Baba Mountains. All the snails were hand-collected. The material is deposited in the private collection of the author (coll. DED.), in the National Museum of Natural History, Sofia (NMNHS), and in the Senckenberg Museum, Frankfurt am Main (SMF). The morphological examinations were carried out with a stereomicroscope.

## Results

### Euxinella alpinella sp. n.

urn:lsid:zoobank.org:act:AD11792A-D992-44E7-B2B9-F3D37DAE59BC

http://species-id.net/wiki/Euxinella_alpinella

Fig. 1

#### Locus typicus.

Republic of Macedonia, Nidzhe Mountains, Belo Grotlo peak, 40°59'17.9'N, 21°49'10.7'E, 2164 m a.s.l., limestone, under stones, 07. June 2010, leg. I. K. Dedov (43 specimens, collected alive, dried). Until now, the new species is known only from the type locality.

#### Type material.

holotype SMF 336340; paratypes: SMF 336341/2 specimens, NMNHS*/* 2 specimens, DED/MK 635/38 specimens.

#### Differential diagnosis.

The new species differs from the two subspecies of *Euxinella radikae* – *Euxinella radikae radikae* and *Euxinella radikae hristovskii* - by occasional presence of a short basalis and the long subcolumellaris, which is visible from outside the aperture. *Euxinella alpinella* sp. n. differs from *Euxinella subaii* by its pale or missing palatal callus, the much shorter or missing basalis, and its shorter palatal plicaes.

#### Description of type series:

shell small, spindle-shaped; shell colour brownish; suture deep; teleoconch striated, finely ribbed on the last whorls of the shell; neck with pronounced basal keel; aperture pear-shaped, in some specimens a slight palatal thickening present situated in parallel to its edge; well developed basal canal, often with a short basalis on its left margin; superior lamella connected with spiralis through a slight depression in the contact zone (or both situated very close, not connected); inferior lamella (columellaris) well developed running steeply nearby parietal side; lunella in dorsal position, sometimes reduced to a pale thickening or short straight plica; principal plica well developed; upper palatalis present, very short; subcolumellar lamella long and visible from outside the aperture, often forming part of the right margin of the basal canal; clausilium partly visible from outside the aperture, oval-orthogonal, distally with a weak edge.

#### Etymology.

This species is named “*alpinella”* because of its isolated type locality in the alpine area.

#### Distribution.

*Euxinella alpinella* sp. n. occurs in open alpine terrains on limestone, up to 2000 m a.s.l. Until now, the species is known only from its type locality, Nidzhe Mountains, in the southern part of the Republic of Macedonia.

#### Ecology.

This species occurs on rocky alpine meadows above the timber line, on limestone rocks and in their crevices and under stones.

#### Comments.

*Euxinella alpinella* sp. n. isnowthe fourth representative of the genus *Euxinella*. It shows shell morphological characters more similar to the forest species *Euxinella subaii*, than to the petrophilous species *Euxinella radikae* (trace of palatal callus, long subcolumellar lamella, basalis present). In *Euxinella radikae*, thesubcolumellaris ends at the level of the lunellar system, which this forms part of the definition of the genus (see also Dedov and Neubert 2009). However, *Euxinella alpinella* sp. n. is the second species which shows differences in this character. As an „alpine form“ (for this term cf. Nordsieck 2008), *Euxinella alpinella* sp. n. shows the highest shell morphological variation among all species of the genus *Euxinella* (different level of reduction of the clausilium aparatus – present or missing of basalis, upper palatal plicae and palatal callus; different intensity of development of lunella – pale thickening or short straight plicae; connected or separated lamella superior and spiralis). The morphological similarity between *Euxinella subaii* and *Euxinella alpinella* indicates some affinities between both taxa, and their particular distribution ranges, the Kozhuf and Nidzhe mountain, are quite close. Probably, both species originate from a parent taxon whose populations have been isolated ecologically (*Euxinella subaii* in deciduous forests, *Euxinella alpinella* sp. n. in alpine mountain meadows and on as well as under rocks). A similar distribution pattern of related species from the Kozhuf and Nidzhe Mountains can even be observed in more mobile organisms such as *Tapinopterus heyrovskii* Jedlicka, 1939 and *Tapinopterus purkynei* Jedlicka, 1928 (Coleoptera: Carabidae), and *Dorcadion heyrovskii* Breuning, 1943and *Dorcadion purkynei* Heirovsky, 1925 (Coleoptera: Cerambycidae) (Hristovski pers. comm.).

**Table 1.** Measurements (in mm) of *Euxinella alpinella* sp. n. and variation of the clausilium apparatus. Abbreviation: H – height of shell , D – diameter of shell , W – number of whorls, We - number of whorls of the protoconch, H_P_ – height of peristome, D_P_ – diameter of aperture, R_1_ – ribs on 1mm of the last whorl. Holotype - №4.

	H	D	W	We	_H_P	_D_P	_R_1	superior+spiralis	basalis
1	10.56	2.64	9	2.5	2.4	1.68	12	connected	lack
2	9.36	2.52	8.5	2	2.28	1.68	10	separated	present
3	9.48	2.64	9	2	2.52	1.56	9	separated	lack
4	10.44	2.64	10	2.5	2.4	1.56	8	connected	present
5	9.84	2.52	9	2	2.4	1.68	9	connected	present
6	9.12	2.4	9	2	2.04	1.56	8	separated	lack
7	9.36	2.64	8.5	2.5	2.52	1.56	11	connected	lack
8	9.6	2.52	9	2.5	2.4	1.68	9	connected	present
9	8.88	2.52	9	2	2.16	1.44	8	separated	lack
10	9	2.52	9	2	2.28	1.56	6	connected	lack
**Average**	**9.56**	**2.56**	**9**	**2.2**	**2.34**	**1.6**	**9**	**--**	**--**
**Variance**	**0.32**	**0.01**	**0.17**	**0.07**	**0.02**	**0.01**	**2.89**	**--**	**--**

##### Key for determination of the species and subspecies of the genus *Euxinella*.

**Table:** 

1(4)	Subcolumellaris ends at the level of the lunellar system. Palatal callus missing.
2(3)	Colour of the shells more yellowish-greenish, basal keel finer, R2 7–18	*Euxinella radikae radikae*
3(2)	Colour of the shells deeper brown, basal keel stronger, R2 21	*Euxinella radikae hristovskii*
4(1)	Subcolumellaris running parallel to basal canal. Palatal callus present.
5(6)	Palatal callus pale or often missing in some extreme forms, basalis shorter or missing	*Euxinella alpinella*
6(5)	Palatal callus well developed, basalis longer	*Euxinella subaii*

**Figure 1.**
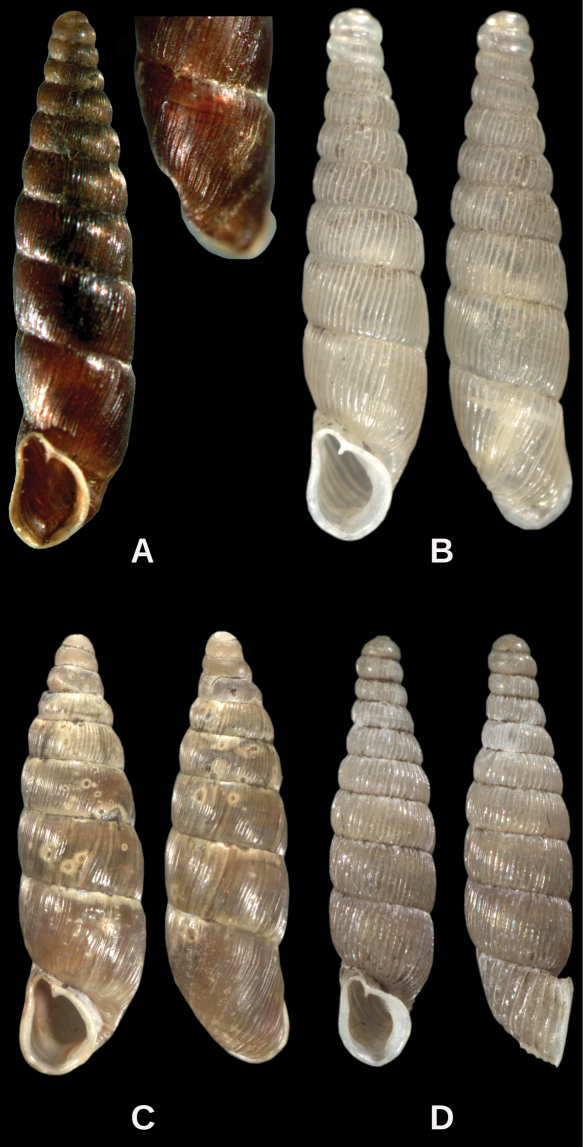
Genus *Euxinella*. **A** *Euxinella alpinella* sp. n. **B** *Euxinella radikae radikae* **C** *Euxinella subaii*; **D** *Euxinella radikae hristovskii*

**Figure 2.**
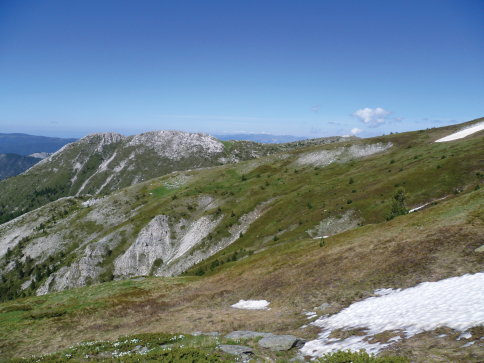
Republic of Macedonia, Nidzhe Mountains, Belo Grotlo peak, type locality of *Euxinella alpinella* sp. n.

### Vestia lazarovii sp. n.

urn:lsid:zoobank.org:act:3338D8CD-0D44-4C78-8511-0198A58E3A10

http://species-id.net/wiki/Vestia_lazarovii

Fig. 3

#### Locus typicu.

Republic of Macedonia, Baba (= Pelister) Mountains near Kopanke hut, 41°01'59.7'N, 21°13'09.0'E, 1639 m a.s.l., *Pinus peuce* forestecotone,under logs of dead wood and fallen trunks, 03. September 2002, leg. S. Lazarov, (2 empty shells); from the same site, 16. June 2009, leg. I. K. Dedov (12 specimens, collected alive, dried).

#### Additional material.

Republic of Macedonia, Pelister (= Baba) Mountains, Palisnopje area, 1450 m a.s.l., *Pinus peuce* forestecotone, under logs and fallen trunks, 16. June 2009, leg. T. Mitev, (2 empty shells).

#### Type material.

holotype SMF 336343, paratypes (n = 13 specimens) SMF 336344/2 specimens; NMNHS/2 specimens*;* DED/MK 453/2 specimens; DED/MK 636/9 specimens; Pelister Mountains, Palisnopje area, 1450 m. a.s.l., DED/MK637/2 specimens).

#### Differential diagnosis.

This species differs from *Vestia roschitzi* (Brancsik, 1890) and *Vestia ranojevici* (Pavlovic, 1912) by the wide spiral turn of its inferior lamella; from *Vestia elata* (Rossmassler, 1836), *Vestia gulo* (E. Bielz, 1859) and *Vestia turgida* (Rossmassler, 1836) by the missing lunella.

#### Description of type series.

shell relatively small, spindle shaped, yellow-brownish coloured; whorls 8.5*–*9.5, including 2*–*2.5 smooth protoconch whorls; teleoconch ribbed (R = 38–54); aperture oval pear-shaped with a whitish, weekly reflected lip; a pale palatal callus present in some specimens; basal canal and keel missing; sinulus wide, not inclined to the shell axis; superior lamella connected with spiralis or close to it; inferior lamella turning widely-spirally; lunella and basalis missing; principal and upper palatal plica usually present; principal plica very short to about 1/3 of the last whorl; upper palatal plica short or missing; clausilium plate varying from hook-shaped in its end as is typical for *Vestia*, or with a weak hook and thin clausilium plate.

#### Etymology.

This species is named after the Bulgarian arachnologist Dr. Stoyan Lazarov-Panagyrsky, B. A. S., Institute of Zoology, who was the first to collect this species.

#### Distribution.

*Vestia lazarovii* sp. n. is currently only known from two sites at 1450 and 1650 m a.s.l. from the Pelister (= Baba) Mountains, Republic of Macedonia.

#### Ecology.

This species occurs in the *Pinus peuce* forest ecotone, under logs of dead wood near Kopanke hut, as well as in the *Pinus peuce* forest ecotone in the Palisnopje area, under logs and fallen trunks.

#### Comments.

The first species of genus *Vestia* to bereported from Macedonia (Urbanski 1960) was *Vestia ranojevici*. Nordsieck (1974) reported it from the Osogovo Mountains, Kalin Kamen area, 1560 m a.s.l., Kriva Palanka district, near to the border with Bulgaria. *Vestia lazarovii* sp. n. is the second representative of the genus from the Republic of Macedonia and occurs relatively high up in the mountains (in coniferous forests and its ecotone) and is characterized by a quite strong reduction of the clausilium apparatus (reduced lunella, short principal and short or missing upper palatal plicae, missing basalis, somethimes very fine and thin clausilium plate with weakly developed hook at its end). A connection between superior and spiral lamellae is typical for the genus *Vestia*, so the specimens with disconnected superior and spiral lamellae could be also interpreted as showing initial reduction in this part of the clausilium apparatus.

**Table 2.** Measurements (mm) of the *Vestia lazarovii* sp. n. and variation of the clausilium apparatus. Abbreviation: H – height of shell, D – diameter of shell, W – number of whorls, We - number of whorls of the protoconch, H_P_ – height of peristome, D_P_ – diameter of aperture, R – ribs on the last whorl. Holotype - №6.

	H	D	W	We	_H_P	_D_P	R	superior+spiralis	hook shape of clausilium
1	9.94	3.00	9	2.5	2.8	2.2	38	connected	prominent
2	9.45	3.1	9	2.5	2.6	2.2	44	separated	broken off
3	10.43	3.2	9	2.5	2.95	2.45	48	separated	not visible
4	9.03	3.1	8	2	2.85	2.05	54	separated	weakly prominent
5	10.43	3.15	9	2.5	2.85	2.35	48	connected	broken off
6	10.64	3.2	9.5	2.5	2.9	2.2	45	connected	prominent
7	10.99	3.25	9.5	2.5	3	2.2	44	separated	weakly prominent
8	10.07	3.05	9	2	2.95	2.1	46	separated	broken off
9	9.73	3.3	9	2	3	2.3	45	separated	prominent
10	9.24	2.95	8.5	2.5	2.9	2.1	42	separated	weakly prominent
**Average**	**10**	**3.13**	**8.95**	**2.35**	**2.88**	**2.22**	**45.4**	**--**	
**Variance**	**0.41**	**0.01**	**0.19**	**0.06**	**0.01**	**0.02**	**17.6**	**--**	

**Figure 3.**
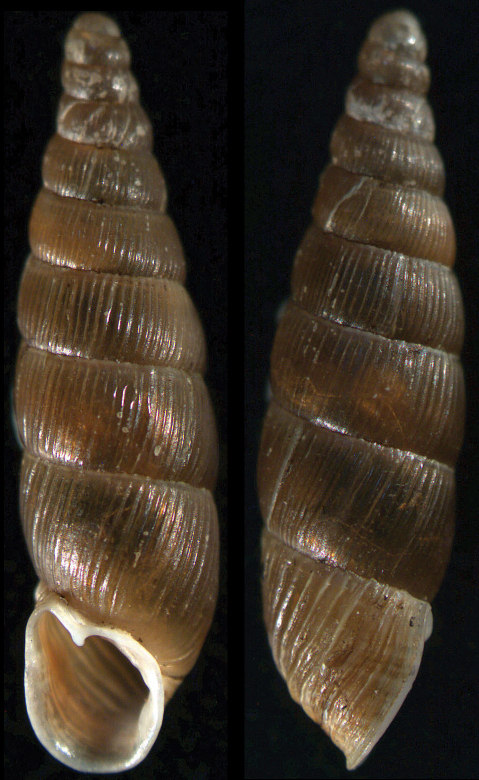
*Vestia lazarovii* sp. n.

## Supplementary Material

XML Treatment for Euxinella alpinella

XML Treatment for Vestia lazarovii
